# Different altered stage correlative expression of high abundance acute-phase proteins in sera of patients with epithelial ovarian carcinoma

**DOI:** 10.1186/1756-8722-2-37

**Published:** 2009-08-27

**Authors:** Yeng Chen, Boon-Kiong Lim, Onn H Hashim

**Affiliations:** 1 Department of Molecular Medicine, Faculty of Medicine, University of Malaya, 50603 Kuala Lumpur, Malaysia; 2 Institute for Research in Molecular Medicine (INFORMM), Universiti Sains Malaysia, 11800 Minden, Penang, Malaysia; 3 Department of Obstetrics & Gynecology, Faculty of Medicine, University of Malaya, 50603 Kuala Lumpur, Malaysia; 4 University of Malaya Centre for Proteomics Research (UMCPR), University of Malaya, 50603 Kuala Lumpur, Malaysia

## Abstract

**Background:**

The general enhanced expression of α_1_-antichymotrypsin (ACT), clusterin (CLU), α_1_-antitrypsin (AAT), haptoglobin β-chain (HAP), and leucine rich glycoprotein (LRG) in the sera of patients with epithelial ovarian carcinoma (EOCa) was recently reported. In the present study, we compared the expression of the serum acute-phase proteins (APPs) in the patients according to their stages of cancer.

**Results:**

Different altered stage correlative expression of the high abundance serum APPs was demonstrated in sera of the patients studied. While the expression of ACT, HAP and AAT appeared to demonstrate positive correlation with the three initial stages of the cancer, inverse correlation was apparently detected in the expression of LRG and CLU. For patients who were diagnosed with stage IV of the cancer, expression of the serum APPs did not conform to the altered progression changes.

**Conclusion:**

Our results highlight the potential prognostic significance of selective high abundance serum APPs in patients with EOCa.

## Background

Epithelial ovarian carcinoma (EOCa) is usually asymptomatic in its early stages and development. For most patients, the disease is often widespread at the time of diagnosis, and this is partly due to the absence of sensitive and reliable serological markers. CA125, the currently accepted serum marker for diagnosis of EOCa, is limited in sensitivity as it is detected in approximately 50% of patients in stage I of the disease, and 80% of women with advanced cancer [1]. Moreover, it lacks specificity as it is also elevated in 30% of nonovarian malignancies, 6% of benign gynecologic disorders, and 1% of normal cases [2]. In addition, several gynecological disorders such as ovarian cysts, uterine leiomyomas, pelvis inflammatory disease and endometriosis produce higher levels of CA125 [3,4].

Advances in proteomics analysis have generated much interest in the prospect of identifying complementary biomarkers for diagnoses of various cancers [5]. Our gel-based proteomic studies performed on unfractionated whole serum samples of patients with different types of cancer had demonstrated the different altered expression of selective serum high abundance acute-phase proteins (APPs) in sera of patients with EOCa [6], germ-line ovarian carcinoma [6], breast cancer [7], nasopharyngeal carcinoma [8], endometrial adenocarcinoma [9], squamous cell cervical carcinoma [9], adenocervical carcinoma [9] and osteosarcoma [10]. In the case of EOCa, enhanced expression of α1-antichymotrypsin (ACT), clusterin (CLU), α1-antitrypsin (AAT) and its fragments (AATf), haptoglobin β-chain (HAP) as well as its cleaved fragments (HAPc) and leucine rich glycoprotein (LRG) was detected in serum samples of the cancer patients compared to control individuals.

In the present study, the expression of the overexpressed high abundance acute-phase proteins (APPs) in sera of the EOCa patients was analysed according to the stages of the cancer.

## Methods

### Serum samples

Serum samples were obtained from newly diagnosed EOCa patients (ages between 24 to 65 years) in different stages of the cancer (stage I, n = 4; stage II, n = 6; stage III, n = 6; stage IV, n = 4) at the University of Malaya Medical Centre (UMMC), prior to treatment. Staging of EOCa was performed in accordance with the International Federation of Gynecology and Obstetrics (FIGO) clinical staging system. Control sera were obtained from 24 age-matched voluntary women without cancer. Samples obtained were with consent and approval granted by the Ethical committee (Institutional Review Board) of the UMMC in accordance with the Declaration of Helsinki and the ICH GCP guideline.

### Two-dimensional gel electrophoresis

Two-dimensional gel electrophoresis (2-DE) was performed as previously described [6-10]. Unfractionated whole human serum samples (10 μl) were subjected to isoelectric focusing in rehydrated precast immobilized dry strips pH 4-7 (GE Healthcare Bio-Sciences, Uppsala, Sweden). Focused samples in the strips were then subjected to electrophoresis using 8-18% gradient polyacrylamide gels in the presence of sodium dodecyl sulfate (SDS-PAGE). All samples were analyzed in duplicate.

### Staining of 2-DE gels

The 2-DE gels were developed by silver staining as described by Heukeshoven and Dernick [11]. For mass spectrometric analysis, gels were stained with Coomassie Blue according to the modified method of Shevchenko *et al*. [12].

### Identification of proteins and database search

Confirmation on the identities of the APP spot clusters by mass spectrometry using the Ettan MALDI-ToF Pro had been previously described [6-9]. Peaklist data obtained from PMF were generated using the Ettan MALDI software (release version 2.0) and 4000 Series Explorer software (release version 3), respectively. The data were exported to the MASCOT search engine (Matrix Science Ltd., London, UK; release version 2.2). The following parameters were used in the search: (i) enzyme: trypsin, (ii) one missed cleavage allowed, (iii) taxonomy limited to *Homo sapiens*, (iv) mass value: monoisotopic, (v) peptide mass tolerance: ± 0.1 Da and (vi) peptide charge state: 1+.

### Image analysis

Images of the 2-DE gels were captured using the LabScan image scanner (Version 5). Protein profiles were evaluated using the ImageMaster™ 2D Platinum Software (Version 5). To eliminate possible variations due to differential protein staining and loading, expression of proteins was evaluated in percentage of volume contribution (%vol), which refers to the volume percentage of a protein taken against the total spot volume of all proteins.

### Statistical analysis

Levels of proteins in the gels are presented as means %vol ± SD of the respective number of samples in each cohort of patients or controls analysed. The Normal test (Z) was used to analyze the significance of differences between control subjects and patients and to examine the correlation between variables. A *P*-value of less than 0.05 (p < 0.05) was considered statistically significant.

## Results

### Serum protein profiles

**Figure 1:**
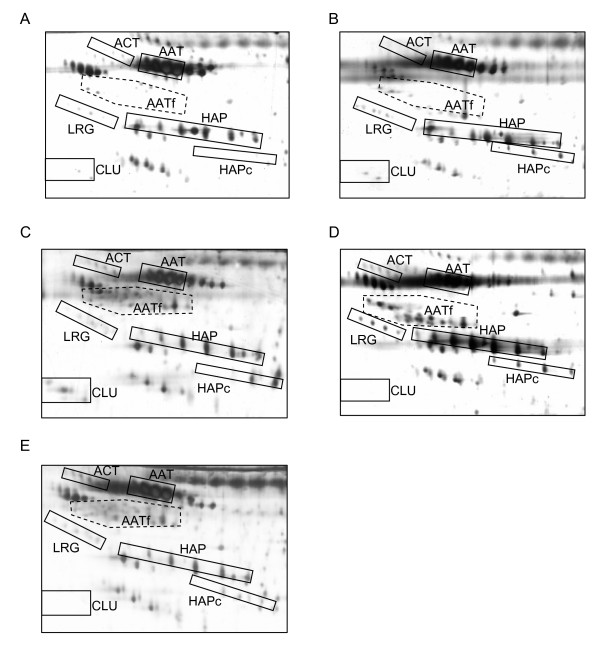
**Typical 2-DE serum protein profiles of negative control women and patients in different stages of EOCa**. Unfractionated serum samples of patients and negative controls were subjected to 2-DE and silver staining. Panel A demonstrates a typical representative 2-DE serum protein profile of negative control women. Panels B, C, D and E demonstrate typical representative unfractionated serum protein profiles of the patients with EOCa in stages I, II, III and IV, respectively. For all panels, the acidic sides of the 2-DE gels are to the left and relative molecular mass declines from the top.

Subjecting unfractionated serum samples of EOCa patients and negative control female subjects to 2-DE and silver staining generated typical highly resolved profiles of the high abundance proteins. Fig. 1 demonstrates the representative 2-DE serum protein profiles of the negative control women (panel A) and EOCa patients in various stages of the disease (panels B-E). Confirmation of the identities of the various serum high abundance APPs had been described in our previous reports [6-9]. ACT, AAT, LRG, CLU and HAP spots clusters were clearly resolved in all profiles. However, HAPc and AATf appeared to be detected only in the sera of the EOCa patients but not in control individuals, while CLU was only detected in sera of patients in stages I and II (panels B and C, respectively), but not those in stages III and IV, and the negative control women.

### Image analysis of 2-DE gels

**Figure 2:**
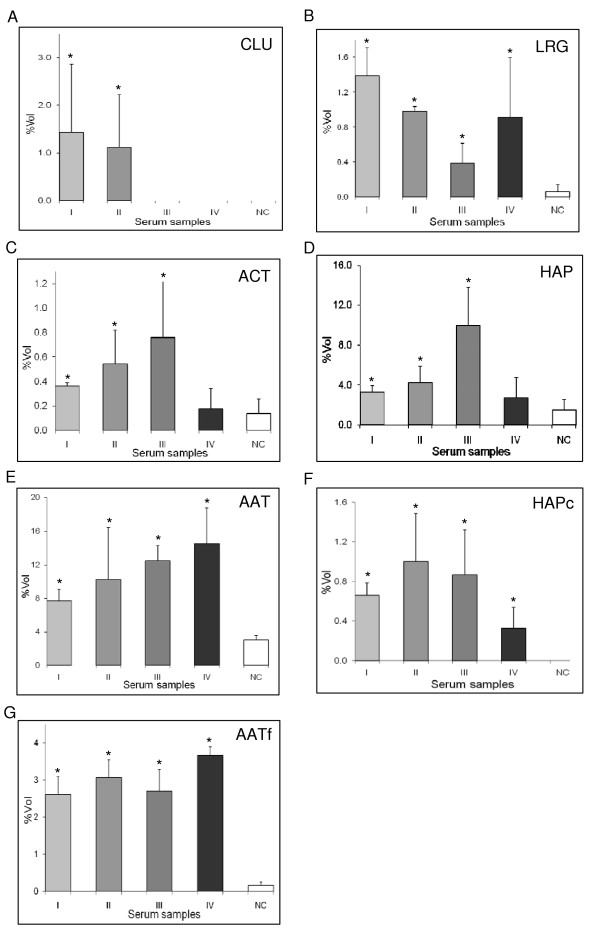
**Mean percentage of volume contribution of APPs expression in negative control women and patients in different stages of EOCa**. Percentage of volume contribution (%vol) of APPs spot clusters were analyzed by ImageMaster™ 2D Platinum Software Version 5. Analysis was performed on the spot clusters of A:CLU, B:LRG, C:ACT, D:HAP, E:AAT, F:HAPc, and G:AATf. I, II, III and IV refer to the various stages of EOCa in patients, while NC refers to the negative control. Results are means ± SD of the respective number of samples in each cohort analysed (stage I, n = 4; stage II, n = 6; stage III, n = 6; stage IV, n = 4; negative control, n = 24). Summary of the relative expression of serum proteins analysed is presented in Table 1. Asterisks denote significantly different values (p < 0.05).

When the clusters of the high abundance serum protein spots from control women and cohorts of patients diagnosed with different stages of EOCa were analyzed by densitometry software, the correlative stage associated expression of the APPs was demonstrated in sera of the patients studied. An inverse correlation with the initial three stages of the cancer was apparently detected in the expression of CLU and LRG (Fig. 2, panels A and B, respectively). However, the expression of ACT, HAP and AAT appeared to be increasing with the first three stages of EOCa (Fig. 2, panels C, D and E, respectively). For patients who were diagnosed with stage IV of the cancer, expression of the serum APPs did not conform to the altered progression changes. The expression of HAPc and AATf did not demonstrate any correlation with all stages of EOCa (Fig. 2, panels F and G, respectively). The relative expressions (fold changes and statistical significance) of the serum high abundance proteins in EOCa patients according to the stage of cancer are summarized in Table 1.

**Table 1:** Relative expression of APPs in the sera of patients in different clinical stages of EOCa.

**Serum proteins**	*Fold changes and statistical significance				**Probability *P***
		
	**Stage I**	**Stage II**	**Stage III**	**Stage IV**	
**CLU**	#	#	nd	nd	0.0004
**LRG**	+23.36-fold; *p *= 0.0001	+16.46-fold; *p *= 0.0001	+6.44-fold; *p *= 0.0001	+15.34-fold; *p *= 0.0128	0.0001
**ACT**	+2.65-fold; *p *= 0.0001	+3.94-fold; *p *= 0.0006	+5.50-fold; *p *= 0.0001	ns	0.2177
**HAP**	+2.24-fold; *p *= 0.0001	+2.84-fold; *p *= 0.0004	+6.78-fold; *p *= 0.0001	ns	0.0001
**AAT**	+2.52-fold;*p *= 0.0001	+3.35-fold;*p *= 0.0001	+4.09-fold;*p *= 0.0001	+4.75-fold;*p *= 0.0001	0.0008
**HAPc**	#	#	#	#	0.0757
**AATf**	+7.30-fold; *p *= 0.0001	+8.91-fold; *p *= 0.0001	+8.12-fold; *p *= 0.0001	+14.14-fold; *p *= 0.0001	0.0388

*Fold change measures the degree of change in protein expression in patients compared to that of negative controls. This is measured by dividing the average spot intensity in the patients by average spot intensity in the control.

# exclusive detection in patients (not detected in negative controls)

ns protein not significantly expressed

nd protein not detected

+ increased in expression

-- decreased in expression

## Discussion

The results of our experiments highlight the potential prognostic significance of several aberrantly expressed APPs, and hence, the need to conduct a study to monitor the expression of the serum proteins with progress of EOCa. In the present study, we demonstrated the correlative expression of ACT, CLU, HAP, AAT and LRG in patients with EOCa in accordance to the three initial stages of the cancer by using the gel-based proteomics approach. However, the stage-correlated expression was not observed for HAPc and AATf, although the protein fragments were generally not detected in the 2-DE protein profiles of normal control women. Unlike the initial three stages of EOCa, the expression of ACT, CLU, HAP, AAT and LRG in sera of EOCa patients in stage IV did not conform to the altered progression changes observed. This was not unexpected, as the APPs response is generally expected to succumb to the malignancy at the final stage of the cancer, and thus affecting their synthesis in the liver. Depending on severity of the malignancy and liver damage in the late stage patients, the levels of the APPs may or may not correlate with the patterns of the altered progression changes.

The correlative expression of high abundance serum APPs that were detected in the present study may be reflective of the acute-phase response of the body at various initial stages of EOCa. The up-regulated expression of CLU in early stages of EOCa may function to suppress the biologically aggressive behavior of the cancer cells and to exert a protective function on surviving cells. CLU has been reported to confer protection against various cytotoxic agents such as UV radiation, heat shock, oxidative stress, TNFα and chemotherapeutic drugs [13-15]. In cases of the non small cell lung and breast cancers, the expression of CLU was demonstrated to be significantly associated with relapse-free and metastasis-free survival of patients [16,17]. Taken together with the data of our present study, this is suggestive of the prognostic significance of CLU in the cancers concerned.

Like CLU, our present study demonstrated that the enhanced expression of LRG appeared to be inversely correlated with progression of EOCa. LRG, whose function is unknown, was consistently elevated in sera of patients with bacterial infections and often increased during viral infections [18]. Patients with severe acute-phase respiratory syndrome were also noted for their enhanced levels of serum LRG [19]. In the case of cancer, increased serum LRG has been observed in patients with liver [20], lung [21] and pancreatic [22] cancers. Together with HAP, it is also one of the few high abundance cancer selective proteins that were identified in a study comparing trypsin-digested peptides of glycoproteins isolated from sera of healthy individuals and lung adenocarcinoma patients [23]. Aside from its potential as a biomarker for diagnosis, the data of our present study further suggests the prognostic value of LRG particularly for EOCa.

ACT is a well-established APP, whose function is primarily associated with inflammation [24]. It's levels have previously been reported to be elevated in sera of patients with EOCa [6], breast cancer [7], and pancreatic cancer [25,26]. Since the serine proteinase inhibitor is known to form a complex with human kallikrein 3 (HK3; also known as prostate-specific antigen) [27], and HK3 has been shown to be produced by a number of tumors including the ovarian tumor [28], it is very likely that the excess ACT detected in sera of the EOCa patients was part of a complex with HK3 or other proteins. However, this could not be verified since the gel-based approach adopted in our studies involved analysis of proteins in their denatured forms.

The expression of HAP appeared to peak in stage III of EOCa. A recent report by Zhao *et al*. also demonstrated that circulating HAP was significantly correlated with the stage of tumor and survival of EOCa patients [29]. These accumulated data imply that the expression of HAP had been affected by the tumor burden. The significant up-regulated expression of HAPc in the sera of EOCa patients in all stages observed in the present study suggests that the cancer associated HAP was rather unstable and prone to proteolysis. However, the generation of HAPc did not appear to correlate with the various stages of EOCa.

Previously reported studies performed on lung, breast and cervical cancer patients have indicated correlative changes in the levels of serum or plasma AAT, especially in the late metastatic stages of the disease [30-32]. This is compatible with the data of our present study. The significant enhanced expression of AAT in patients with EOCa in all stages also provides explanation to the detection of its cleaved fragment spots, AATf, exclusively in samples from patients. The lower molecular weight AATf was likely generated by proteolytic digestion of the abundant AAT within the cancer microenvironment. Like HAPc, however, cleavage of the serum protein did not appear to correlate with the stages of EOCa.

## Conclusion

The different altered stage correlative expression of CLU, LRG, ACT, HAP and AAT in sera of patients with EOCa was demonstrated in the present study. Our results highlight the potential prognostic significance of the high abundance serum APPs in patients with EOCa.

## Competing interests

The authors declare that they have no competing interests.

## Authors' contributions

YC carried out the experiments and analyzed the data. BKL provided the serum samples for the study and shared his clinical expertise on epithelial ovarian carcinoma. OHH conceptualized the study, designed it and authored the manuscript. All authors read and approved the final manuscript.
